# Lamb Wave-Minimum Sampling Variance Particle Filter-Based Fatigue Crack Prognosis

**DOI:** 10.3390/s19051070

**Published:** 2019-03-02

**Authors:** Weibo Yang, Peiwei Gao

**Affiliations:** College of Civil Aviation, Nanjing University of Aeronautics and Astronautics, Nanjing 210016, China; gpw1963@nuaa.edu.cn

**Keywords:** prognostics and health management, fatigue crack growth, piezoelectric transducer, particle filter, minimum sampling variance, Lamb wave

## Abstract

Fatigue cracks are one of the common failure types of key aircraft components, and they are the focus of prognostics and health management (PHM) systems. Monitoring and prediction of fatigue cracks show great application potential and economic benefit in shortening aircraft downtime, prolonging service life, and enhancing maintenance. However, the fatigue crack growth process is a non-linear non-Gaussian dynamic stochastic process, which involves a variety of uncertainties. Actual crack initiation and growth sometimes deviate from the results of fracture mechanics analysis. The Lamb wave-particle filter (LW-PF) fatigue-crack-life prediction based on piezoelectric transducer (PZT) sensors has the advantages of simple modeling and on-line prediction, making it suitable for engineering applications. Although the resampling algorithm of the standard particle filter (PF) can solve the degradation problem, the discretization error still exists. To alleviate the accuracy decrease caused by the discretization error, a Lamb wave-minimum sampling variance particle filter (LW-MSVPF)-based fatigue crack life prediction method is proposed and validated by fatigue test of the attachment lug in this paper. Sampling variance (SV) is used as a quantitative index to analyze the difference of particle distribution before and after resampling. Compared with the LW-PF method, LW-MSVPF can increase the prediction accuracy with the same computational cost. By using the minimum sampling variance (MSV) resampling method, the original particle distribution is retained to a maximum degree, and the discretization error is significantly reduced. Furthermore, LW-MSVPF maintains the characteristic of dimensional freedom, which means a broader application in on-line prognosis for more complex structures.

## 1. Introduction

As a common failure model of mechanical damage, fatigue cracks account for 50% to 90% of the total failures and are one of the most dominant and most dangerous form of structural damage [1]. The initiation and propagation of fatigue cracks are affected by many uncertainties, such as the statistical features at the microscale of materials, material parameters, machining errors, internal damage, loading, stress ratio, temperature, etc. Fatigue cracks are the result of synergies of inevitability and contingency, that is, basic rules of fatigue cracks and various objective or cognitive uncertainties. Many researchers have studied the physical uncertainties and statistical models [2,3,4]. However, due to the inefficient combination with monitoring methods, the traditional fatigue crack life prediction theory and fatigue test data cannot efficiently update prediction results in real time to achieve early warning and maintenance.

Prognostics and health management (PHM) has been a research hotspot in recent years [5], its goal is to improve the combat effectiveness, agility, accuracy, and sustainability of modern aircraft in a more cost-effective manner. Life prediction, as an indispensable part of PHM technology, is of great significance to prolong the flight life [6], reduce the downtime, and formulate the maintenance strategies.

For fatigue crack life prediction, particle filter (PF)-based prognosis method [7] has combined the advantages of crack propagation law, finite element modeling (FEM), PF, and on-line monitoring technology. Its modeling is relatively simple and does not require a large amount of experimental and training data, which indicates a powerful tool for fatigue crack growth prediction under uncertainties [8]. Moreover, the application of Lamb wave active on-line monitoring technology can realize the on-line prognosis [8,9], which makes the Lamb wave-particle filter (LW-PF)-based prognosis method more suitable for engineering applications.

Based on the benefits of PF, Orchard et al. [10,11] proposed an on-line prediction system for crack life prediction of UH-60 planetary gear by using the PF algorithm. Compared with Kalman filtering, the PF-based method has higher prediction accuracy and can correct the inaccuracy during the monitoring process in real time. However, as a monitoring method for crack growth, its observation equation accuracy can be further developed. Cadini and Zio et al. [12,13] used the simulation data in reference [14] to realize the fatigue crack life prediction via standard PF. Corbetta et al. [15] presented a dynamic state–space model for fatigue-induced structural degradation, which was verified by the standard PF with the simulation data of a central crack specimen. Then, the proposed method was applied to the hole-edge crack growth life prediction of helicopter siding [16], where a vernier caliper was used as the observation method. Meanwhile, Corbetta et al. pointed out that the complexity of the state–space model and stress intensity factor (SIF) were main factors that affected the realization of on-line prediction. An et al. [17] provided a practical review of neural networks, Gaussian process regression, PF, and the Bayesian method for prognostics. It has been concluded that the PF and Bayesian methods are more suitable for fatigue crack life prediction, but these two methods can only be applied when the physical model and boundary conditions are known. Chen and Yang et al. [9,18] established the state–space model based on Paris law and Lamb wave active health monitoring. By using the standard PF method, the on-line prediction of crack growth on edge and central cracked aluminum plate were realized, influences brought by uncertainties of crack propagation parameters and monitoring methods were effectively eliminated, and a higher prediction accuracy was achieved. To overcome the sample impoverishment problem of the standard PF, Yuan and Yang et al. [8,19] proposed a Lamb wave-deterministic resampling particle filter (LW-DRPF) method for fatigue crack prognosis, and it was verified on attachment lugs and edge cracked aluminum plate during fatigue tests. The prediction results of LW-PF and LW-DRPF indicate that the prediction accuracy has been further improved by LW-DRPF. However, since the deterministic resampling algorithm loses the property of dimensional freedom, the LW-DRPF method is only applicable in state–space models of the second-order or below, while it fails to be applied to higher-order models. To improve the importance function, Chen et al. [20] substituted the transfer probability density function with the mixture of measurement and transition probability density function. Then, he drew out a Gaussian weight–mixture proposal particle filter method for on-line prognosis of fatigue crack propagation. The validation result of attachment lugs shows the effectiveness of the dynamic updated process for the state equation and the prediction accuracy is higher than that of standard PF. Li et al. [6] employed a genetic algorithm to improve the particle leanness problem, and the algorithm performed better than the traditional PF algorithm and support vector machine (SVM) in remaining useful life (RUL) predictions of a rolling element bearing. Jouin et al. [21] reviewed 46 references of prognostics works based on PF, which included crack growth, Li-ion batteries, turbine blades (creep growth), bearings, Jet engines, and so on. They summarized that most of the current applications were based on standard PF and needed more effective experimental verification.

In summary, the above-mentioned research of PF based fatigue crack life prediction are still in infancy, and most of them are based on standard PF and applied to simple specimens. Although standard PF can solve the degeneracy problem by the resampling method, it introduces the sample impoverishment at the same time [22], that is, the particle set will lose its diversity after several iterations, which inevitably decreases the prediction accuracy. One way to settle the sample impoverishment is to ensure the consistency of particle distribution before and after resampling, that is, the sample times of the *i*^th^ particle should be equal to its mathematical expectation (particle number multiplied by weight $Nw_{t}^{i}$) [23]. However, since the sample times should be an integer, the discretization error inevitably exists in particle distribution after resampling.

The simplest way to eliminate the discretization error and conquer the disadvantage of sample impoverishment is to increase the particle number [24]. However, this solution will greatly increase the computation cost and affect the realization of on-line prediction. In order to mitigate the particle impoverishment caused by the discretization error and improve the prediction accuracy of crack growth life prediction, sampling variance (SV) is used as a metric to study the difference of particle distribution before and after resampling in this paper. Based on the analysis results of SV, an on-line prediction method of Lamb wave-minimum sampling variance particle filter (LW-MSVPF) is proposed and validated by fatigue test of an attachment lug. The comparison of life prediction results shows that the SV of LW-MSVPF is smaller than that of LW-PF with particle number being the same, which can preserve the particle distribution to a maximum degree before and after resampling. Meanwhile, LW-MSVPF has the same computational complexity as LW-PF, that is to say, it can increase the prediction accuracy without increasing computation cost.

The organization of this paper is as follows. Section 2 introduces the essential state–space model of the fatigue crack growth, which includes the evolution equation based on Paris law and the observation equation based on the active Lamb wave crack monitoring method. In Section 3, the details of the on-line crack growth prediction method based on LW-MSVPF are illustrated. Then, the upper proposed method is verified by fatigue tests of attachment lug specimens in Section 4. Finally, Section 5 gives the conclusion.

## 2. State–Space Model for Fatigue Crack Growth

The dynamic stochastic process of fatigue crack growth is the process of crack size evolving with time according to the crack propagation laws. This kind of process, which evolves with time according to determining or statistical laws, can be completely described by the state–space model. The state–space model includes an evolution equation that describes the crack growth and an observation equation that describes the crack monitoring, as shown in Equation (1).

(1) $$\{\begin{array}{c} x_{t+1}=f(x_{t},\omega_{t})\\ z_{t+1}=g(x_{t+1},\upsilon_{t+1}) \end{array}$$

where *x_t_* is the crack length at time *t*, f(⋅) is defined by a crack growth model, through which the crack growth law from time *t* to *t* + 1 is stated, $\omega_{t}$ is a random variable denoting the uncertainty during crack growth, $z_{t+1}$ is the observation value at *t* + 1 which represents the observation value corresponding to the crack length monitored by the active Lamb wave based monitoring method, g(⋅) is the relationship between the observation value and the crack length, and $\upsilon_{t+1}$ represents the uncertainties during monitoring.

The particle filter has the functions of smoothing, filtering, and predicting. The application for structural fatigue crack growth life prediction is based on the crack status values from 0 to *t* and the crack observations from 1 to *t*. Then, the state–space model and crack-observation update at *t* + 1 are used to predict the crack status value at time *t* + 1 and beyond. Finally, the RUL of the structure can be calculated by the predicted crack status value (such as crack size) to determine whether the structure can continue to be used or not.

### 2.1. Evolution Equation

At present, the Paris law [25] is often used to construct the evolution equation to describe the law of crack propagation, as shown in Equation (2).

(2) $$\frac{da}{dN_{f}}=C{\left(\Delta K\right)}^{m}$$

where *a* is the crack length, *N*_f_ is the number of loading cycles, *C* and *m* are material parameters, and Δ*K* refers to the SIF range, which can be found in the SIF handbook [26] or calculated by the finite element method (FEM) [9]. However, the handbook is only suitable for some simple specimens. On the other hand, although FEM can be applied to complex specimens, it requires high computational resources and can only be used off-line. The application range of both methods is limited. To reduce calculation consumption and extend the application range of SIF, this paper uses a simplified version of the SIF range [19,27], as shown in Equation (3).

(3) $$\Delta K=\Delta \sigma \sqrt{\pi a}$$

where Δ*σ* is the stress amplitude.

If Equation (2) is integrable, the fatigue crack life prediction can be realized. However, the above integral is not easy to achieve in reality. Therefore, Equation (2) is often approximated in the form of a difference equation when the fatigue load time interval Δ*N*_f_ is small enough. The law of crack propagation is shown in Equation (4).

(4) $$a_{t+1}\approx a_{t}+\frac{da_{t}}{dN_{f}}\Delta N_{f}=a_{t}+C{\left(\Delta K\right)}^{m}\Delta N_{f}$$

where $a_{t+1}$ is the crack size at time *t* + 1, and Δ*N*_f_ is the discrete step of loading cycles.

To take the uncertainty during fatigue crack growth into consideration, Zio et al. [13] adopted the following revised model, as shown in Equation (5).

(5) $$\frac{da}{dN_{f}}=10^{\omega_{C}(t)}C{\left(\Delta K\right)}^{m}$$

where $\omega_{C}(t)$ is a zero-mean additive white Gaussian noise that follows $N(0,\sigma_{\omega_{C}}^{2})$, denoting the uncertainty of material parameters during crack propagation, and $\sigma_{\omega_{{}_{C}}}$ is the standard deviation [18,19,20]. The results of a large number of fatigue tests show that material parameters *C* and *m* have differences even with the same type of structure under the same working conditions, which characterizes the uncertainty of crack growth. The distribution $\omega_{C}(t)$ of the material parameter *C* can be calculated by the secant method [28] via results from multiple groups of fatigue tests. Taking the logarithm of both sides of Equation (2), Equation (6) is obtainable.

(6) $$\log\left(\frac{da}{dN_{f}}\right)=\log C+m\log\left(\Delta K\right)$$

where log*C* and *m* are intercept and slope, respectively.

Perform *Q*-1 group fatigue tests for a certain type of structure and label the specimens as ${\left\{J_{l}\right\}}_{l=1}^{Q-1}$. Collect *M* groups of crack observations ${\left\{a_{t}^{l}\right\}}_{t=1}^{M}$ for *l*^th^ specimen, and the crack growth rate can be approximated by its differential form of Equation (7) at time $\left[({\left\{N_{f}^{l}\right\}}_{t+1}+{\left\{N_{f}^{l}\right\}}_{t})/2,\;(a_{t+1}^{l}+a_{t}^{l})/2\right]$.

(7) $${\left(\frac{\Delta a}{\Delta N_{f}}\right)}_{\frac{t+1+t}{2}}^{l}=\frac{a_{t+1}^{l}-a_{t}^{l}}{{\left\{N_{f}^{l}\right\}}_{t+1}-{\left\{N_{f}^{l}\right\}}_{t}}$$

Substitute *M* groups of crack observations into Equation (7) to calculate the crack growth rate at each moment, then substitute them into Equation (2) to obtain material parameters log*C* and *m* by linear regression [29]. After that, logarithmic transformation of log*C* and *m* help to obtain material parameters $C^{l}$ and $m^{l}$ for *l*^th^ specimen. Then, assign the parameters to *C*, *m,* and $\omega_{C}(t)$ [8,9] in Equation (5), as shown in Equation (8).

(8) $$C=\mathrm{mean}({\left\{C^{l}\right\}}_{l=1}^{Q-1}),\;m=\mathrm{mean}({\left\{m^{l}\right\}}_{l=1}^{Q-1}),\;\omega_{C}(t)=N(0,\mathrm{Var}({\left\{\log C^{l}\right\}}_{l=1}^{Q-1})$$

where mean(·) is the mean function, and Var(·) the variance function. In Equation (8), material parameter *C* and state noise $\omega_{C}(t)$ can be merged into *C_t_*. Meanwhile, taking the other uncertainty during fatigue crack growth into consideration, an additive zero mean Gaussian white noise of $\omega_{t}\sim N\left(0,\sigma_{\omega }^{2}\right)$ can be added to build the evolution equation [10,11,30], as showed in Equation (9).

(9) $$\left\{ \begin{array}{l} a_{t+1}=a_{t}+C_{t}{\left(\Delta K\right)}^{m}\Delta N_{f}+\omega_{t}\\ C_{t+1}=C_{t} \end{array}\right.$$

There are two noise distributions in Equation (9), which consider not only the uncertainties of the material parameters in the Paris law but also other uncertainties during fatigue crack growth. Combined with particle filter, these two noise distributions in Equation (9) have better robustness in an application.

### 2.2. Observation Equation

#### 2.2.1. *Lamb Wave-Based Fatigue Crack On-line Monitoring Method*

**Figure 1:**
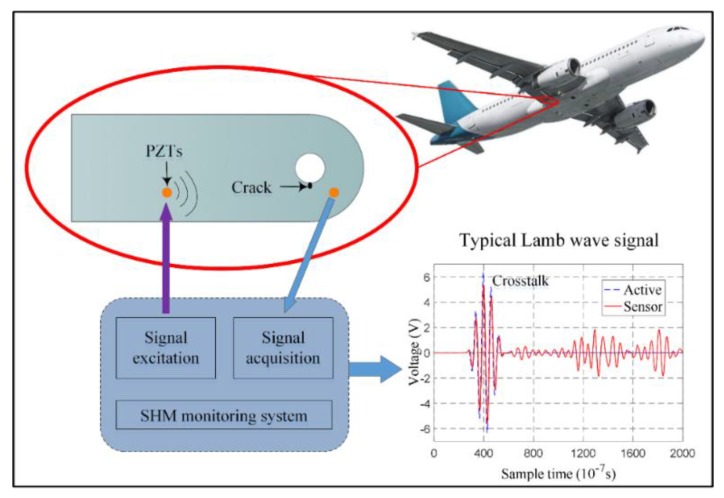
Active Lamb wave on-line crack monitoring method.

**Figure 2:**
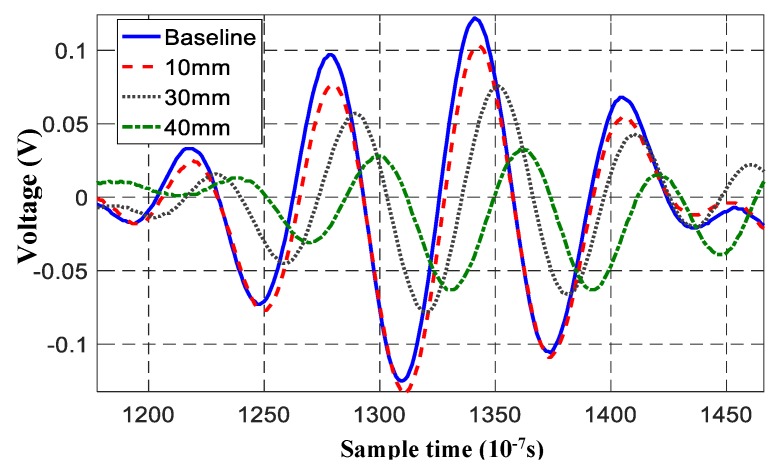
Response signal changes under different crack lengths.

Piezoelectric transducer (PZT)-based active Lamb wave method of structural health monitoring (SHM) is adopted for crack monitoring in this paper [31,32,33,34,35,36]. Figure 1 gives a typical configuration used for crack monitoring. Once the Lamb wave is excited in the structure by a PZT, the response signal will be influenced by the crack growth. Figure 2 gives typical signal changes during the crack growth, which are mainly focused on phase and amplitude. Specific damage index (*DI*) can be extracted for quantifying the crack length by comparing the response signals under healthy and cracked conditions. To capture these changes, the first arrival wave packet, named the direct wave packet, is used to calculate the *DI*, named the scattering signal normalized energy (SSNE) [33] in this paper, as shown in Equation (10).

(10) $$DI=\sqrt{\frac{\int_{t_{1}}^{t_{2}}{\left|D\left(t\right)-H\left(t\right)\right|}^{2}dt}{\int_{t_{1}}^{t_{2}}{\left|H\left(t\right)\right|}^{2}dt}}$$

where *D*(*t*) is the response signal since the crack growth started, *H*(*t*) is the baseline signal acquired when the structure is healthy, and $t_{1}$ and $t_{2}$ are the start and end sampling times of the first wave packets received by the PZT sensors. The first received wave could be S_0_ mode, A_0_ mode or the mixed wave packet of both, which depends on the frequency of excitation signal, specimen size, sensor layout, and so on. SSNE *DI* shows the scattering signal energy changes along with crack size, in which the scattering signal is obtained by comparing response signals between crack growth and the healthy condition.

#### 2.2.2. Lamb Wave-Based Observation Equation

To establish the relationship between crack length and observed *DI*, an observation equation is adopted in this paper. With a set of specimens being tested in advance, the data driven method is used to model the observation equation as Equation (11):

(11) $$z_{t+1}=g(a_{t+1})+\upsilon_{t+1}$$

where $g(a_{t+1})$ is a polynomial function, and $\upsilon_{t}\sim N(0,\sigma_{\upsilon }^{2})$ refers to observation uncertainties of the active Lamb wave-based method.

The specific solution process of $g(a_{t+1})$ is as follows: If *Q*-1 specimens of a fatigue test were carried out in a certain structural type, label them as ${\left\{J_{l}\right\}}_{l=1}^{Q-1}$. For each specimen, collect *M* groups of response signal data at crack size of ${\left\{a_{t}^{l}\right\}}_{t=1}^{M}$ via active Lamb wave on-line monitoring method. Analyze the response signals by a specific *DI* algorithm to obtain *Q*-1 groups of *DI* monitoring results on-line, mark them as $\left\{DI_{t+1}^{l},t=0\ldots M-1,l=1\ldots Q-1\right\}$. Polynomial fitting is employed to establish the relationship between *DI* and crack length, as shown in Equation (12), then use it to evaluate the monitoring results of the *Q*^th^ specimen.

(12) $$DI_{t+1}=b_{0}+b_{1}a_{t+1}+b_{2}a_{t+1}^{2}+b_{3}a_{t+1}^{3}+\ldots b_{n}a_{t+1}^{n}$$

where $b_{0}{,b}_{1}{,b}_{2}{,b}_{3}\ldots b_{n}$ are coefficients of the polynomial fitting.

Assuming the measurement uncertainty is in compliance with a zero mean Gaussian white noise of $\upsilon_{t}\sim N(0,\sigma_{\upsilon }^{2})$, the standard deviation $\sigma_{\upsilon }$ is close to the root-mean-square error (RMSE) of the polynomial fitting. Therefore, the observation equation is achieved as Equation (13).

(13) $$DI_{t+1}=b_{0}+b_{1}a_{t+1}+b_{2}a_{t+1}^{2}+b_{3}a_{t+1}^{3}+\ldots b_{n}a_{t+1}^{n}+\upsilon_{t+1}$$

## 3. LW-MSVPF-Based Fatigue Crack Growth Prognosis

### 3.1. Standard PF

The essence of standard PF-based fatigue crack growth prognosis is to realize the unbiased estimation of crack growth by Bayesian estimation, as shown in Equation (14).

(14) $${\hat{x}}_{t+1}=E({\hat{x}}_{t+1}^{-})={\hat{x}}_{t+1}^{-}p(x_{t+1}|z_{1:t+1})$$

where superscript “^” represents posteriori estimate, superscript “-” priori estimate, and *p*(*x_t_*_+1_|*z*_1:*t*+1_) is the probability density function (PDF) with the observations of *z*_1_, *z*_2_,…*z_t_*_+1_. Since *p*(*x_t_*_+1_|*z*_1:*t*+1_) is modified by observation *z_t_*_+1_ at time *t* + 1, it is often called posterior PDF, and *p*(*x*_0:*t*+1_|*z*_1:*t*+1_) is the edge probability density of *p*(*x_t_*_+1_|*z*_1:*t*+1_), their relationship can be defined as Equation (15).

(15) $$p(x_{t+1}|z_{1:t+1})=\int \int \ldots \int p(x_{0:t+1}|z_{1:t+1})dx_{0}\ldots dx_{t}\Rightarrow \int p(x_{0:t+1}|z_{1:t+1})dx_{0:t}$$

where symbol ⇒ denotes the simplification of multiple integrals.

Assuming that the crack growth satisfies the first-order Markov process [24], *p*(*x_t_*_+1_|*z*_1:*t*+1_) can be obtained by Bayesian estimation iterations as shown in Equation (16).

(16) $$\{\begin{array}{l} p(x_{t+1}|z_{1:t})=\int p(x_{t+1}|x_{t})p(x_{t}|z_{1:t})dx_{t}\\ p(x_{t+1}|z_{1:t+1})=\frac{p(z_{t+1}|x_{t+1})p(x_{t+1}|z_{1:t})}{p(z_{t+1}|z_{1:t})} \end{array}$$

Therefore, when the initial state of *x*_0_ and priori PDF *p*(*x*_0_|*z*_0_) are given, Equation (16) can recur *p*(*x_t_*|*z*_1:*t*_) to *p*(*x_t_*_+1_|*z*_1:*t*+1_) from time *t* to *t* + 1, afterwards, state prediction of crack propagation from *t* + 1 to later can be reached. However, the multi-integral in Equation (16) is hard to calculate in practice. Hammersley et al. [37] introduces the Monte Carlo simulation method to solve this problem: sampling *N* independent identically distributed (i.i.d.) samples from *p*(*x*_0:*t*+1_|*z*_1:*t*+1_), when *N*~∞, samples’ mean is approximated to mathematical expectations according to the Wiener–Khinchin theorem of large numbers as Equation (17).

(17) $${\hat{x}}_{t+1}=E({\hat{x}}_{t+1}^{-})={\hat{x}}_{t+1}^{-}p(x_{t+1}|z_{1:t+1})\approx \frac{1}{N}\sum_{i=1}^{N}{\hat{x}}_{t+1}^{-}(i)$$

Whereas, it is hard to sample from *p*(*x_t_*_+1_|*z*_1:*t*+1_) practically, an important density *q*(*x*_0:*t*+1_|*z*_1:*t*+1_) is introduced to the Monte Carlo simulation method, which is not only easy to sample but also identically distributed as *p*(*x_t_*_+1_|*z*_1:*t*+1_), as shown in Equation (18).

(18) $$E({\hat{x}}_{t+1}^{-})=\int {\hat{x}}_{t+1}^{-}\frac{p(x_{0:t+1}|z_{1:t+1})}{q(x_{0:t+1}|z_{1:t+1})}q(x_{0:t+1}|z_{1:t+1})dx_{0:t}$$

Using the Monte Carlo simulation method, Equation (18) can be simplified to Equation (19).

(19) $$E({\hat{x}}_{t+1}^{-})=\frac{\int {\hat{x}}_{t+1}^{-}{\tilde{w}}_{t+1}q(x_{0:t+1}|z_{1:t+1})dx_{0:t}}{\int {\tilde{w}}_{t+1}q(x_{0:t+1}|z_{1:t+1})dx_{0:t+1}}\approx \frac{\frac{1}{N}\sum_{i=1}^{N}{\hat{x}}_{t+1}^{-}(i){\tilde{w}}_{t+1}^{i}}{\frac{1}{N}\sum_{i=1}^{N}{\tilde{w}}_{t+1}^{i}}=\sum_{i=1}^{N}{\hat{x}}_{t+1}^{-}(i)w_{t+1}^{i}$$

where $w_{t}^{i}$ is the normalization particle weight, the weight normalization is also given in Equation (20), and the non-normalized particle weight ${\tilde{w}}_{t}^{i}$ can be derived into an iterative form.

(20) $$w_{t+1}^{i}=\frac{{\tilde{w}}_{t+1}^{i}}{\sum_{i=1}^{N}{\tilde{w}}_{t+1}^{i}},{\tilde{w}}_{t+1}={\tilde{w}}_{t}\frac{p(z_{t+1}|x_{t+1})p(x_{t+1}|x_{t})}{q(x_{t+1}|x_{0:t},z_{1:t+1})}$$

The derivation process above is called sequential importance sampling (SIS). Via SIS, the unbiased estimation of crack propagation can be expressed by the weighted summation of priori estimation and their corresponding weights. Nevertheless, since the optimal solution of $q(x_{t+1}^{i}|x_{t}^{i},z_{1:t+1})=p(x_{t+1}^{i}|x_{t}^{i},z_{1:t+1})$ is difficult to solve, SIS is confronted with a serious degeneracy problem [24]. To overcome this degeneracy phenomenon, in 1993 Gordon et al. [7] proposed a scheme of sub-optimal solutions $q(x_{t+1}^{i}|x_{t}^{i},z_{1:t+1})=p(x_{t+1}^{i}|x_{t}^{i})$ combined with a multinomial resampling method, which is named sequential importance resampling (SIR), also called PF. As a result, the weight updating can be simplified as Equation (21).

(21) $${\tilde{w}}_{t+1}^{i}={\tilde{w}}_{t}^{i}\cdot p(z_{t+1}|x_{t+1}^{i})$$

With the application of the SIR method, the posterior estimation of crack length is calculated by the weighted summation of priori estimation and their corresponding weights, as shown in Equation (22).

(22) $${\hat{a}}_{t+1}=\sum_{i=1}^{N}a_{t+1}^{i}w_{t+1}^{i}$$

Using the iteration process, the fatigue crack growth prognosis can be performed as shown in Equations (23) and (24)

(23) $$a_{t+d}^{i}=a_{t+d-1}^{i}+C_{t+d-1}^{i}{\left(\Delta K\right)}^{m}\Delta N+\omega_{t+d-1}$$

(24) $${\hat{a}}_{t+d}=\sum_{i=1}^{N}a_{t+d}^{i}w_{t+1}^{i}$$

Although SIR can solve the degeneracy problem by multinomial resampling, it introduces the side effect of sample impoverishment [22], which means that the particle set will lose its diversity after several iterations and this will lead to a prediction accuracy decrease [7,38,39]. One way to address the particle impoverishment is to ensure the consistency of particle distribution before and after resampling, that is, the sample times of *i*^th^ particle should be equal to its mathematical expectation (particle number multiplied by weight $Nw_{t}^{i}$) [23]. However, since the sample times should be an integer, a discretization error inevitably exists after resampling in the particle distribution.

### 3.2. Minimum Sampling Variance Resampling

To reduce the side effects of sample impoverishment and increase prediction accuracy, a cost function of SV is introduced to provide metrics for differences in discrete distributions before and after particle resampling [23], as shown in Equation (25).

(25) $$SV=\frac{1}{U}\sum_{u=1}^{U}{\left(N_{t}^{\left(u\right)}-Nw_{t}^{\left(u\right)}\right)}^{2}$$

where *U* is the particle number under different state values after resampling, and it characterizes the diversity of particles, $N_{t}^{\left(u\right)}$ is the actual resample times of the particle and its value should be an integer, and $Nw_{t}^{\left(u\right)}$ is the expectation of particle resampling times.

Under the metrics of SV, the minimum sampling variance (MSV) can relieve the sample impoverishment to the maximum extent after resampling. In order to minimize SV after resampling, multinomial resampling proposed by Gordon et al. [7] should satisfy the condition that all particle weights are equal to 1/*N* as Equation (26).

(26) $$w_{t}^{i}=1/N$$

Equation (26) is an optimal condition, and the degeneracy problem can be perfectly overcome when it is satisfied. Under the optimal condition, the solving of MSV becomes an integer programming problem [40]. Based on the objective of finding the MSV, an MSV resampling method is proposed by Li et al. [40]. The flow of the MSV resampling is as follows:

(1) Copy particles: For *i*^th^ particle, if its particle weight is bigger than 1/*N*, copy this particle for $n_{i}$ times, where $n_{i}$ is the integer part of the product, as Equation (27).
  (27) $$n_{i}=\mathrm{Floor}(Nw_{t+1}^{i})$$
  where Floor(·) is the function rounded towards minus infinity. When the copy process ends, a copy particle set of ${\left\{x_{t+1}^{i},\right\}}_{i=1}^{L}$ is obtained, and all of their corresponding particle weights are equal to 1/*N*. The total number of the copy particle set is $N_{L}=\sum_{i}n_{i}$.
(2) Residual particles: After extracting copy particles from the original particle set ${\left\{x_{t+1}^{i}\right\}}_{i=1}^{N}$, the residual particle weights can be calculated by Equation (28). The set composed of residual particles and their residue weights ${\left\{x_{t+1}^{i},{\overset{⌢}{w}}_{t+1}^{i}\right\}}_{i=1}^{N}$ are called residual particle set.
  (28) $${\overset{⌢}{w}}_{t+1}^{i}=\frac{N{\tilde{w}}_{t+1}^{i}-\left\lfloor N{\tilde{w}}_{t+1}^{i}\right\rfloor }{N}$$
(3) MSV resampling: Sort the residual particle set according to their residue weights, then sample *N*-*L* particles with the largest particle weights. The process above can be characterized by the function of TopRank*_N_*_-*L*_(·), as shown in Equation (29).
  (29) $${\left\{x_{t+1}^{i}\right\}}_{i=1}^{N-L}={\mathrm{TopRank}}_{N-L}({\left\{x_{t+1}^{i},{\overset{⌢}{w}}_{t+1}^{i}\right\}}_{i=1}^{N})$$
(4) Update the particle set: Add *N*-*L* MSV particles to the copy particles to restore the total particle number to *N*, then update the new particle set as ${\left\{x_{t+1}^{i},w_{t+1}^{i}\right\}}_{i=1}^{N}$, where all particle weights of $w_{t+1}^{i}$ are equal to 1/*N*.

Under the conditions of 1 ≤ *u* < *U* and $\left|N_{t}^{u}-Nw_{i}^{u}\;\right|<1$, the MSV resampling preserves the posterior PDF to the largest extent, which means the least information loss during resampling. When *N*~+∞, the MSV resampling method is asymptotically unbiased [23].

### 3.3. On-Line Fatigue Crack Growth Prognosis Based on LW-MSVPF

**Figure 3:**
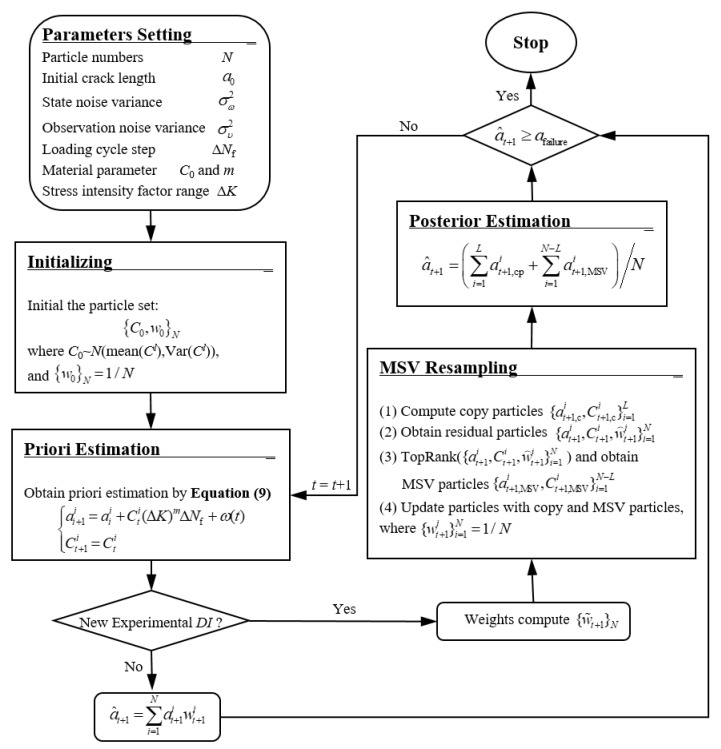
Lamb wave-minimum sampling variance particle filter (LW-MSVPF) flow chart of fatigue crack growth prognosis.

Figure 3 shows the flow chart of LW-MSVPF based fatigue crack growth prognosis. 

First, prepare the parameters setting and initialization modules for LW-MSVPF, which should be finished before on-line crack prognosis. Second, monitor the crack on-line by PZT based active Lamb wave method during the fatigue crack growth. Then, during the prognosis procedure, obtain the priori estimation first by Equation (9) for a certain time interval. When a new *DI* is monitored on-line during the prognosis procedure, use it to calculate the particle weights and perform MSV resampling to obtain the posterior estimation of the crack length. When there is no new *DI* detected during the procedure, the posterior estimation is output directly by using Equation (30). Repeat the upper prediction step sequentially until ${\hat{a}}_{t+1}\geq a_{\mathrm{failure}}$, where $a_{\mathrm{failure}}$ represents a pre-set failure crack length, and the total time intervals are the remaining useful life of the structure.

(30) $${\hat{a}}_{t+1}=\left(\sum_{i=1}^{L}a_{t+1,\mathrm{cp}}^{i}+\sum_{i=1}^{N-L}a_{t+1,\mathrm{MSV}}^{i}\right)/N$$

where $a_{t+1,\mathrm{cp}}^{i}$ and $a_{t+1,\mathrm{MSV}}^{i}$ are posterior crack estimations after the step of copy particles and MSV resampling, respectively.

## 4. Experimental Evaluation

### 4.1. Experimental Setup

The attachment lug is a critical joint type in aircraft structures, for it is often used as a normal connection in wing girders, fuselages, engines, and so on [41]. During service, between the interface of the pin and lug, the attachment lug suffers from cyclic loading and fretting, which will induce crack initiation and growth. Moreover, when transmitted through pins or bolts, tensile load in lugs will cause wear on the contact surfaces due to compression and friction. Uncertainties of crack growth will increase under this wear phenomenon.

**Figure 4:**
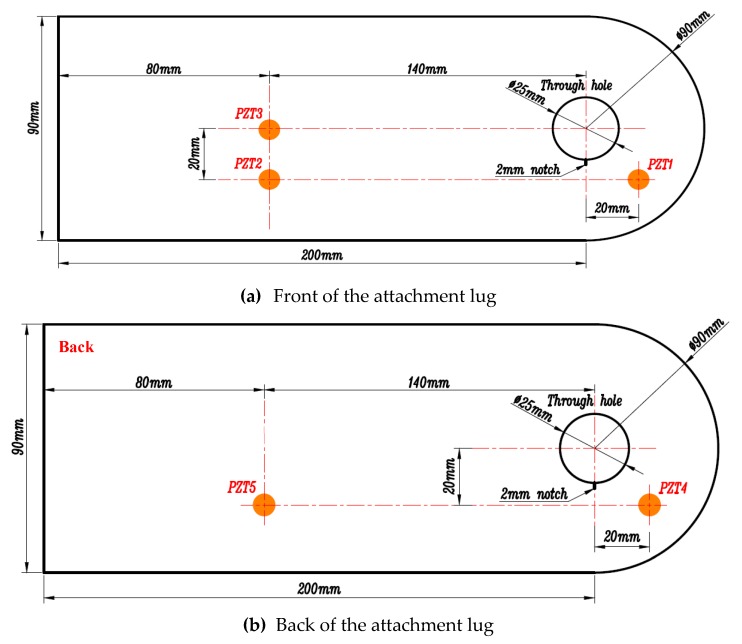
Attachment of lug crack specimens and sensor layout. (**a**) Front of the attachment lug; (**b**) Back of the attachment lug.

In total, seven fatigue tests were performed on attachment lugs to validate the proposed method. The dimensions of each test piece were the same. These specimens were manufactured using 5 mm thick LY12 aluminum and with a 25 mm diameter hole, and were labeled from CT1 to CT7. Attachment lug crack specimens and sensor layouts are shown in Figure 4. During service, stress concentration usually happens at the side edge of the hole according to the finite element analysis. Since crack initiation is most likely to happen at the stress concentration area, a 2 mm notch was machined at the edge of the lug hole to initiate the crack and control the direction of the crack growth.

**Figure 5:**
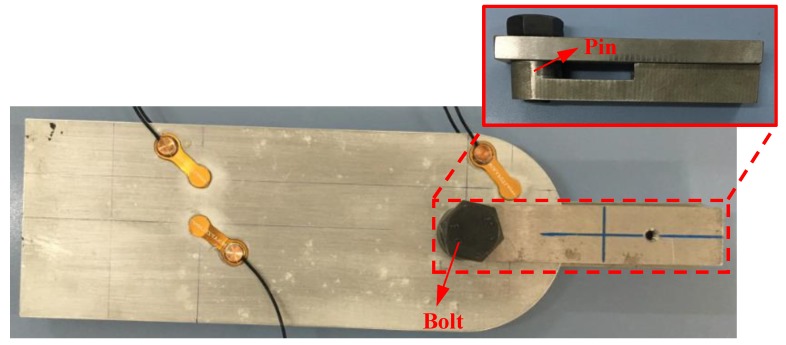
Attachment of lug specimen and the corresponding dowel pin fixture.

**Figure 6:**
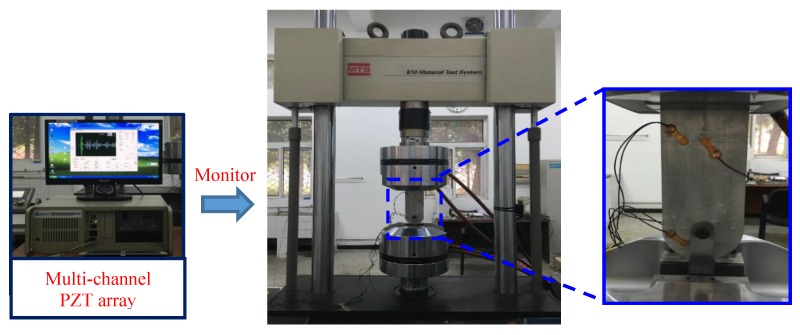
The experimental setup.

Figure 5 shows the attachment lug and fixture. To install the lug specimen, a dowel pin was adopted to connect it and the designed fixture, which transmits the axial tension load to the attachment lug. This is a real load-transmitting style in engineering applications. Figure 6 shows the experimental setup. Fatigue load was applied by using a MTS810 electro-hydraulic servo tensile machine. The active Lamb wave-based monitoring was performed by employing the multi-channel PZT array scanning system [42]. In the experiment, the excitation signal, a 3-cycle tone-burst signal with 160 kHz central frequency and ±70V amplitude, was chosen, and its frequency thickness product was 800 kHz·mm [8]. Sampling frequency of the Lamb wave was 50 MHz. During the experiment, specimen CT1 was used for a static test without sensors, and specimens CT2–CT7 were used for fatigue tests. Five sensors were laid on the lug and labelled from PZT1 to PZT5, three of which were arranged on the front and two on the back. PZT1 was used as the actuator, PZT2 and PZT3 were applied as sensors that formed two monitoring channels named channel 1−2 and 1−3 separately. The SSNE damage indices (*DI*s) calculated by Equation (10) show that channel 1−2 has a better relation between *DI*s and crack lengths [8]. Channel 4−5 was formed by PZT4 and PZT5, which was symmetrically arranged on the other side of the specimen and had similar performance to channel 1−2, and was used to assess the repeatability of experimental data.

**Figure 7:**
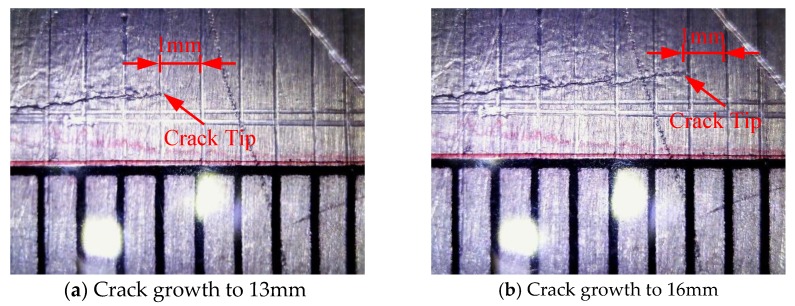
Direct measurement of the crack performed by electronic magnifier. (**a**) Crack growth to 13 mm; (**b**) Crack growth to 16 mm.

A tensile test of specimen CT1 was first performed to determine the fatigue load. The fracture load was obtained as 72 kN. According to this fracture load, a sinusoidal load with a peak value of 18 kN, which was 25% of the fracture load, was chosen for CT2–CT7 specimens. The stress ratio was *R* = 0.1 and a 10 Hz loading frequency was adopted. During the fatigue test, the direct measurement of the crack was performed by an electronic magnifier. When 1 mm interval crack growth was monitored, the fatigue testing would be suspended and held to the peak load of 18 KN for measurement by the PZT based on the active SHM system [42]. Figure 7 shows the actual crack length measured by electronic magnifier.

### 4.2. State–Space Model for Attachment Lug

**Figure 8:**
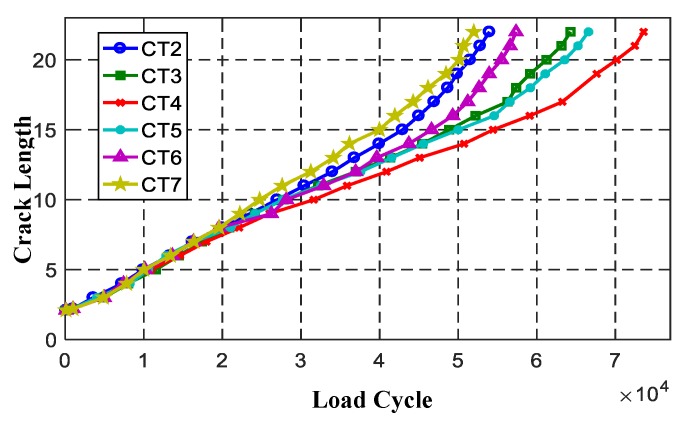
Crack growth of specimens CT2–CT7.

**Figure 9:**
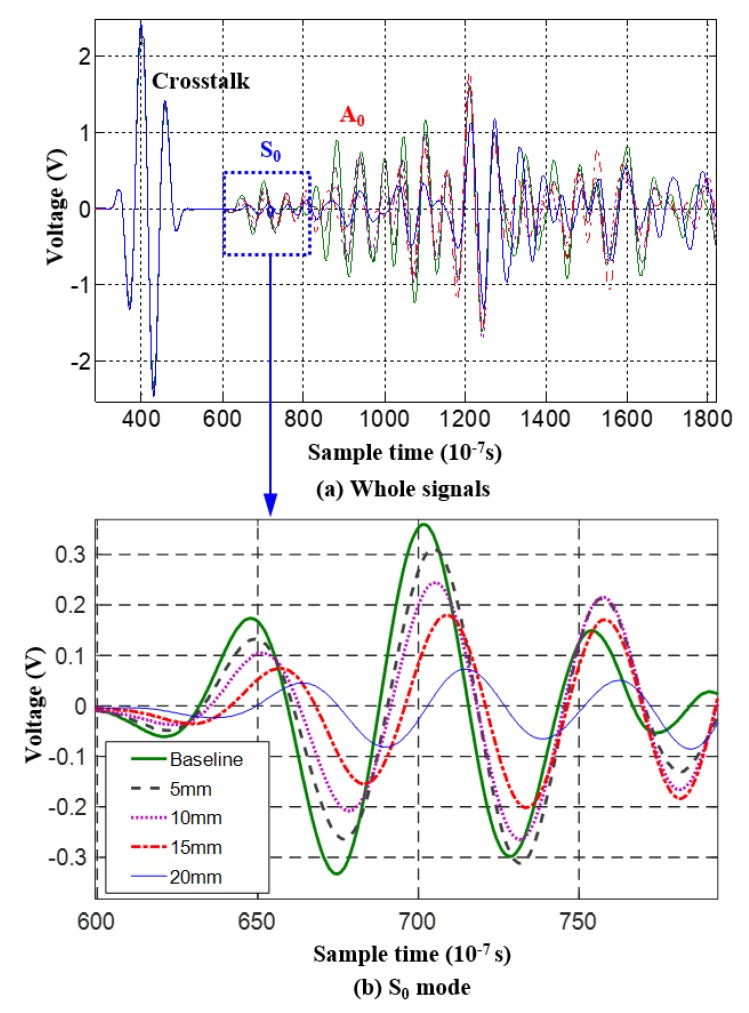
Response signal changes with crack growth (CT2): (**a**) Whole signals; (**b**) S_0_ mode.

Crack growth processes of CT2–CT7 are shown in Figure 8. Obvious uncertainties can be found during the fatigue crack growth trajectories, especially when the crack size is larger. Figure 9 is the response signal changes with crack growth (CT2).

In order to verify the feasibility of the proposed method in this paper, monitoring results obtained from CT2–CT6 specimens were employed to establish the state–space model, and CT7 was used for prognosis validation. Using the method described in Section 2.1, material parameters $\log C_{0}$ and *m* of CT2–CT6 specimens were calculated in advance according to the crack growth data of Figure 8. And material parameters calculated are given in Table 1. The distribution of $\log C_{0}$ and *m* were obtained as $\log C_{0}\sim N\left(-7.6084,0.2172^{2}\right)$, *m* = 0.84, then applied into Equation (9) to construct the evolution equation, where the time interval was set as $\Delta N_{f}=60$.

**Figure 10:**
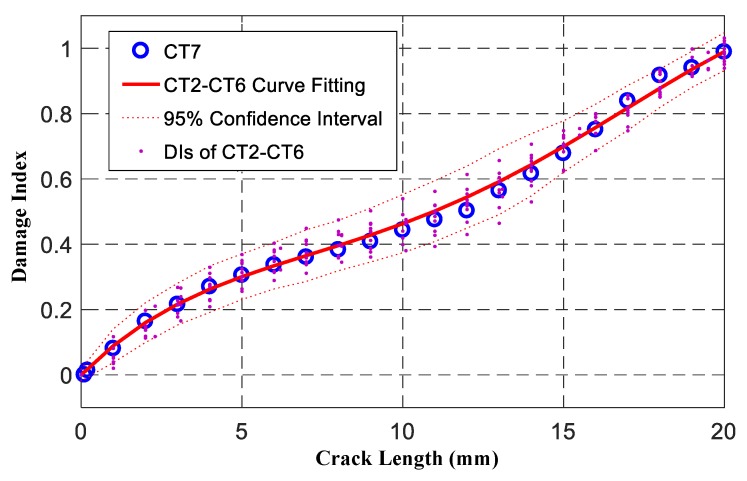
Comparison of CT7 experimental *DI*s and observation equation fitting curves.

The response signals of CT2–CT6 under different crack length were analyzed by the SSNE *DI* of Equation (10). Then *DI*s data and crack length of CT2–CT6 specimens were used to fit the observation equation using the curve fitting tool of Matlab, as shown in Equation (31). According to the RMSE of the fitting curve, which was 0.0397, the observation uncertainty was set as $\upsilon_{t+1}\sim N\left(0,0.0397^{2}\right)$. Figure 10 shows the comparison of CT7 experimental *DI*s and observation equation fitting curves. It can be found from Figure 10 that all CT7 experimental *DI*s were in the 95% confidence interval and the max error was −0.0420, which verifies the correctness of the constructed observation equation.

(31) $$\begin{array}{ll} DI_{t+1}= & -1.545\times 10^{-5}a_{t+1}^{4}+7.468\times 10^{-4}a_{t+1}^{3}-0.01127a_{t+1}^{2}+\\ & 0.09978a_{t+1}^{}+1.64\times 10^{-17}+\upsilon_{t+1} \end{array}$$

With the evolution and observation equations having been constructed, the state–space model of Equation (32) was established.

(32) $$\left\{ \begin{array}{l} a_{t+1}=a_{t}+C_{t}{\left(\Delta K\right)}^{m}\Delta N_{f}+\omega_{t}\\ C_{t+1}=C_{t}\\ DI_{t+1}=-1.545\times 10^{-5}a_{t+1}^{4}+7.468\times 10^{-4}a_{t+1}^{3}-0.01127a_{t+1}^{2}+\\ \;0.09978a_{t+1}^{}+1.64\times 10^{-17}+\upsilon_{t+1} \end{array}\right.$$

where material parameters *C_t_* of each particle were initialized as a random sample from $\log C_{0}\sim N\left(-7.6084,0.2172^{2}\right)$, and *m* was initialized as the mean of 0.8400. The state noise was set as $\omega_{t}\sim N\left(0,0.044^{2}\right)$ and the observation noise $\upsilon_{t+1}\sim N\left(0,0.0397^{2}\right)$. Standard deviation $\sigma_{\omega }$ of the state uncertainty, which was twice the maximum average crack growth rate of the CT2–CT6 specimens, was defined as $\sigma_{\omega }=0.044$. The initial crack length was $a_{0}=2\;mm$ and crack lengths of all particles were initialized as $a_{0}$. During the prognosis of specimen CT7, particle number was *N* = 100. Load cycle step was set to be $\Delta N_{f}=60$.

### 4.3. On-Line Fatigue Crack Growth Prognosis

**Figure 11:**
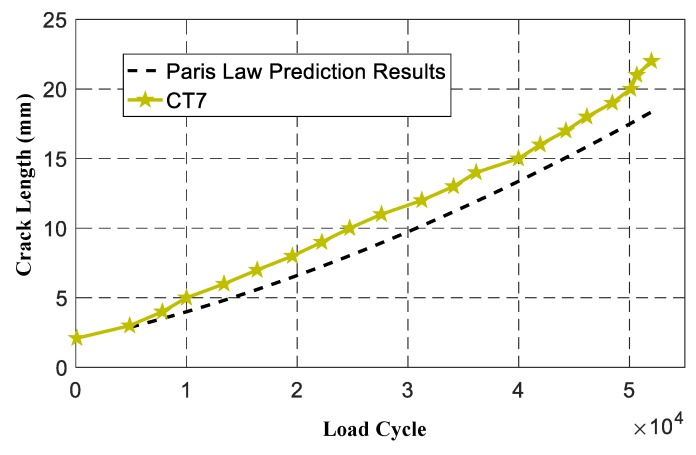
Comparison of CT7 experimental crack growth and Paris law prediction results.

CT7 was adopted to evaluate the proposed on-line crack prognosis method based on LW-MSVPF. When CT7 crack initiation observation was monitored, crack growth of CT7 was first prognosed only by the evolution equation which was described by the Pairs law. Where Δ*K* was calculated by FEM, and material parameters *C* and *m* were set as the mean of specimens CT2–CT6 by the secant method [28], as shown in Table 2. The comparison of CT7 experimental crack growth and the Paris law prediction results are shown in Figure 11. The results show that the physical model summarized by the Pairs law is poor for prognosis, because it fails to take account of the uncertainties during crack growth [2,3].

**Figure 12:**
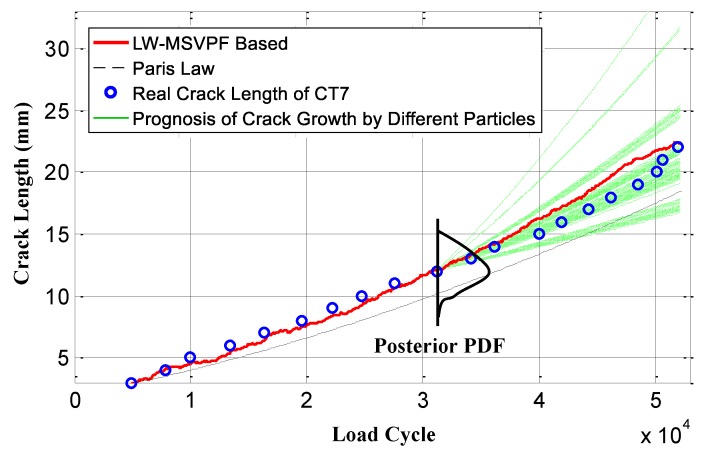
Process of the LW-MSVPF-based crack growth prognosis.

By applying the proposed on-line LW-MSVPF method, observation of the real crack length was taken into account. Each time a monitoring process was performed, a new *DI* was obtained. The LW-MSVPF used different particles to represent the uncertainties of crack growth, and the new obtained *DI* was adopted to determine the particle weights so as to obtain the posterior PDF of the crack length. This posterior PDF is viewed as the prognosis crack length. Figure 12 shows this process.

**Figure 13:**
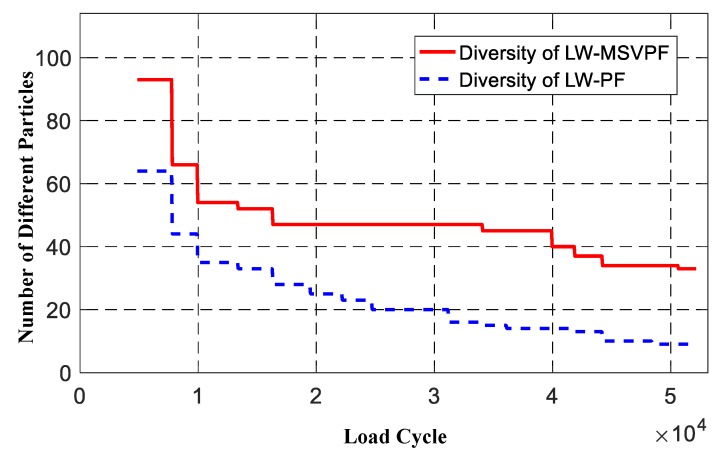
Comparison of particle diversity in the LW-MSVPF and Lamb wave-particle filter (LW-PF).

**Figure 14:**
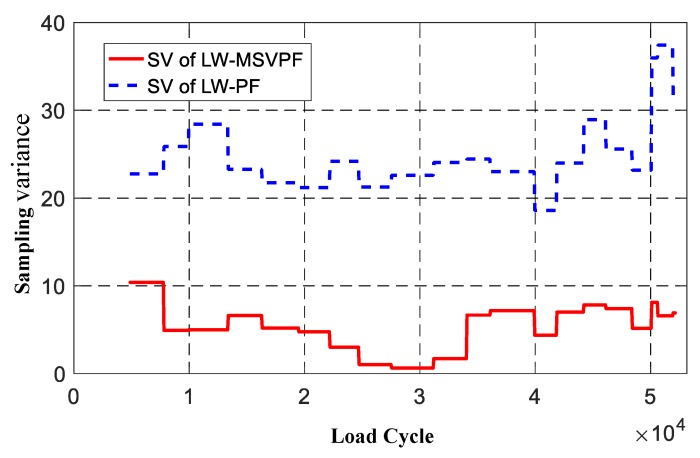
Comparison of the sampling variance (SV) in the LW-MSVPF and LW-PF.

Using the number of particles with different values in ${\left\{C_{t+1}^{i}\right\}}_{i=1}^{N}$ to represent particle diversity, the particle diversity of the LW-PF and LW-MSVPF-based prognosis methods are compared in Figure 13. It can be found that the LW-MSVPF-based method can mitigate the sample impoverishment problem during the prognosis since its particle diversity stays at a significantly higher level than that of LW-PF; when the prognosis is over, the survived particle number was only 9 for LW-PF but 33 for LW-MSVPF. Using SV as a quantitative index to analyze the difference of particle distribution before and after resampling, results of LW-MSVPF and LW-PF are shown in Figure 14. It can be seen from Figure 14 that the SV of LW-MSVPF was significantly lower than that of LW-PF and the means were 5.2011 and 24.2240, respectively. After optimization of the MSV resampling algorithm, discretization error before and after resampling was significantly reduced, and the original distribution of particles was retained to a maximum degree.

**Figure 15:**
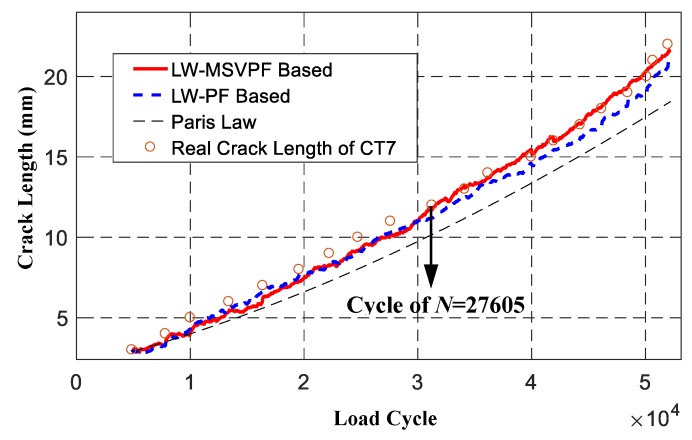
Prognosis results of the LW-MSVPF and LW-PF considering all observations.

Figure 15 shows the prognosis results given by the LW-PF and LW-MSVPF-based methods. It can be found that, when loading cycle *N* was less than 27,605, prognosis accuracy of both methods were similar. However, when loading cycle *N* was greater than 27,605, sample impoverishment of the LW-PF was serious, which led to the decrease of prognosis accuracy. Since the proposed LW-MSVPF prognosis method mitigated the sample impoverishment, its prognosis accuracy was maintained at a high level. Compared with the LW-PF method, the RMSE of the LW-MSVPF method decreased from 0.8516 to 0.6022, demonstrating the superiority of the proposed LW-MSVPF prognosis method. Calculation times were 0.4262 s and 0.4302 s, respectively (MATLAB R2016b, 64-bit 8G memory, Core i5-3230M 2.60GHz, Win7 system), which means that the LW-MSVPF method can obtain a higher precision with the same computational cost than the LW-PF. Although the LW-MSVPF method has not overcome the sample impoverishment problem and is slightly less accurate than the LW-DRPF method [8,19], it has not lost the characteristic of dimensional freedom, which means a broader application in on-line prognosis for more complex structures.

## 5. Conclusions

This paper proposes an on-line Lamb Wave-Minimum Sampling Variance Particle Filter-based crack prognosis method. Verification on the attachment lug specimen showed that the proposed LW-MSVPF can realize an on-line prognosis by adopting the MSV resampling method, and the sample impoverishment problem met by standard PF can be mitigated as well, which increases the prediction accuracy with the same computational cost. The original distribution of particles is retained to a maximum degree, and the discretization error before and after resampling is significantly reduced. Compared with LW-DRPF, LW-MSVPF maintains the characteristic of dimensional freedom, which means a broader application in on-line prognosis for more complex structures.

Although LW-MSVPF has been proved to be effective, the sample impoverishment problem still exists. How to effectively retain the original distribution of particles calls for further study. Meanwhile, evaluations for more complex geometry, loading conditions under different stress ratios, and non-constant amplitudes also need to be performed for further improvement of the proposed method.

## Figures and Tables

**Table 1:** The material parameters log*C*_0_ and *m* of CT2–CT6 specimens.

Specimen	CT2	CT 3	CT 4	CT 5	CT6	Mean	Variance
log*C*_0_	−7.7759	−7.5975	−7.3793	−7.3684	−7.9209	−7.6084	0.2172
*m*	1.0083	0.8222	0.6144	0.6386	1.1163	0.8400	0.1982

**Table 2:** Material parameters log*C* and *m* of specimens CT2–CT6 (Δ*K* is calculated by FEM).

Specimen	CT2	CT3	CT4	CT5	CT6	Mean	Variance
log*C*	−21.5201	−21.1103	−19.4788	−19.3082	−25.0831	−21.3001	−21.5201
*m*	11.1345	10.7800	9.5322	9.4383	13.7625	10.9295	11.1345
